# Identification of quantitative polymerase chain reaction reference genes suitable for normalising gene expression in the brain of normal and dystrophic mice and dogs

**DOI:** 10.12688/wellcomeopenres.16707.1

**Published:** 2021-04-16

**Authors:** Abbe H. Crawford, John C. W. Hildyard, Dominic J. Wells, Richard J. Piercy

**Affiliations:** 1 Clinical Science and Services, Royal Veterinary College, London, UK; 2 Comparative Biomedical Sciences, Royal Veterinary College, London, UK

**Keywords:** qPCR, normalisation, dystrophin, mouse, dog, brain

## Abstract

**Background:** In addition to progressive, debilitating muscle degeneration, ~50% of patients with Duchenne muscular dystrophy (DMD) have associated cognitive and behavioural disorders secondary to deficiency of dystrophin protein in the brain. The brain expresses a variety of dystrophin isoforms (Dp427, Dp140 and Dp71) whose functions remain to be fully elucidated. Detailed comparative analysis of gene expression in healthy and dystrophin-deficient brain is fundamental to understanding the functions of each isoform, and the consequences of their deficiency, with animal models representing a key tool in this endeavour. Reverse transcription quantitative real-time PCR (RT-qPCR) is a widely used method to study gene expression. However, accurate quantitative assessment requires normalisation of expression data using validated reference genes. The aim of this study was to identify a panel of suitable reference genes that can be used to normalise gene expression in the brain of healthy and dystrophic dogs and mice.

**Methods:** Using the DE50-MD dog and *mdx* mouse models of DMD we performed RT-qPCR from fresh frozen brain tissue and employed the geNorm, BestKeeper and Normfinder algorithms to determine the stability of expression of a panel of candidate reference genes across healthy and dystrophic animals, and across different brain regions.

**Results:** We show that *SDHA, UBC* and *YWHAZ* are suitable reference genes for normalising gene expression in healthy and dystrophic canine brain, and *GAPDH, RPL13A* and *CYC1* in healthy and dystrophic murine brain. Notably, there was no overlap in the highest performing reference genes between the two species.

**Conclusions:** Our findings suggest that gene expression normalisation is possible across six regions of the canine brain, and three regions of the murine brain. Our results should facilitate future work to study gene expression in the brains of normal and dystrophic dogs and mice and thus decipher the transcriptional consequences of dystrophin deficiency in the brain.

## Introduction

Duchenne muscular dystrophy (DMD) is the most common lethal genetic disorder diagnosed in childhood, with a worldwide incidence of 1:3500–5000 male births ( Mah *et al.*, 2014). This muscle degenerative condition is caused by an absence or severe deficiency of the sarcolemma-associated protein dystrophin, leading to fragile muscle fibres that sustain injury under physiological use. Affected boys have progressive muscle weakness and typically require a wheelchair before their teens. The disease also affects the heart and respiratory muscles, leading to cardiac and respiratory failure usually by the mid-late twenties ( Rall & Grimm, 2012).

**Figure 1.**
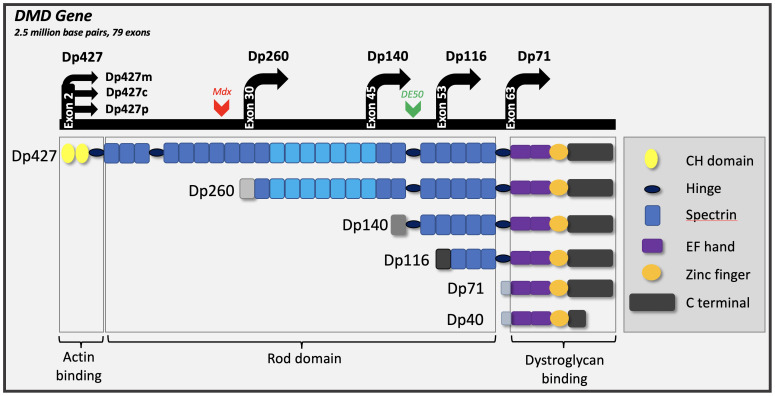
Schematic depiction of the dystrophin gene and its transcripts. Seven promoters are identified within the dystrophin gene; three giving rise to full-length isoforms (Dp427c,m,p) and four internal promoters generating shorter isoforms (Dp260 (retinal isoform), Dp140, Dp116 (Schwann cell isoform) and Dp71). Alternate splicing of Dp71 results in the production of a further isoform (Dp40). Dp427 has multiple functional domains, subsets of which are retained by the shorter isoforms. The additional actin-binding motif of Dp427 and Dp260 isoforms is indicated with lighter shading of spectrin repeats 11–17. The site of the *mdx* mouse mutation in Exon 23 is depicted, and of the DE50-MD dog mutation in exon 50.

Dystrophin is also expressed in the brain. The gene has multiple isoforms named according to their size, arising from internal promoters: muscle expresses only the largest isoform (Dp427m), but expression within the brain is more diverse, including additional full-length dystrophins (Dp427c and p) and the shorter isoforms, Dp140 and Dp71 ( Figure 1). Mutations within the dystrophin gene may affect one or more isoforms. As a consequence of brain dystrophin deficiency, approximately 50% of DMD patients have neurodevelopmental and cognitive disorders, including difficulties with emotional and behavioural regulation (such as anxiety, disordered conduct, low mood) and neurodevelopmental or psychological problems (including intellectual impairment, attention deficit/hyperactivity disorder, autism spectrum disorder) ( Banihani *et al.*, 2015; Hendriksen & Vles, 2008; Pane *et al.*, 2013). These disorders have a major negative impact on the ability of DMD patients to lead fulfilling and independent lives and add additional stresses to affected families and to healthcare systems.

Increasingly, efforts are being made to elucidate the precise expression patterns and functional roles of dystrophin with the brain. Such studies are often dependent on animal models, and several models of DMD are available, including mouse ( Bulfield *et al.*, 1984), rat ( Larcher *et al.*, 2014), dog ( Kornegay, 2017; Walmsley *et al.*, 2010) and pig ( Klymiuk *et al.*, 2013). Models vary in terms of their severity and progression of muscle pathology, cost, therapeutic tractability ( Wells, 2018) and mutational locus ( Im *et al.*, 1996). While mouse models have historically been the most commonly studied, dogs have a disease phenotype that more closely mirrors that of human DMD patients in both severity and progression at the muscle level. Canine models are thus increasingly utilised in DMD translational research ( Amoasii *et al.*, 2018; Barraza-Flores *et al.*, 2019; Le Guiner *et al.*, 2017) but the consequences of dystrophin deficiency within the brain in such models remains less thoroughly characterised in many aspects, including gene expression.

Reverse transcription quantitative real-time PCR (RT-qPCR) is a sensitive and accessible technique to determine the extent and potential significance of changes in gene expression: it is especially suited to directed assessment of transcriptional changes, rather than more global RNAseq approaches. Its utility in brain tissue is enormous, with the potential to study changes in transcription with ageing and disease, and to identify key disease-associated genes. The accuracy of RT-qPCR is critically dependent upon effective internal normalisation as the processing of tissue to RNA and then cDNA entails various stages, each of which may vary in efficiency and so dramatically affect subsequent quantification. Furthermore, as gene expression levels can vary between and within tissues (particularly with ageing and in disease states ( Bustin, 2000; Coulson *et al.*, 2008; Suzuki *et al.*, 2000; Warrington *et al.*, 2000)), normalisation of data with reference genes that exhibit stable expression under the conditions studied is essential. However, evidence suggests that frequently, no single gene is suitable in all scenarios and many reference genes show marked changes in expression between tissues and between disease states ( Bustin, 2002; Butterfield *et al.*, 2010; Chapman & Waldenstrom, 2015; Dheda *et al.*, 2004; Maruyama *et al*, 2001). An ideal panel of reference genes would remain valid independent of brain region studied or whether the sample in question was healthy or dystrophic.

The minimum information for publication of quantitative real-time PCR experiments (MIQE) guidelines describe the importance of reference gene selection in RT-qPCR ( Bustin *et al.*, 2009), highlighting that all studies should use at least two reference genes, each validated for the conditions studied. Previous approaches to identification of suitable reference genes *de novo* have entailed evaluation of expression of multiple candidate genes, in multiple representative cDNA samples, with comparative metrics utilised to identify the most stable. Commonly-used algorithms include geNorm ( Vandesompele *et al.*, 2002a) and Bestkeeper ( Pfaffl *et al.*, 2004a), both of which use pairwise, correlation-based approaches, as well as Normfinder ( Andersen *et al.*, 2004b) that assesses expression stability of individual candidates. Reliance on a single method can be prone to bias but where a candidate gene demonstrates strong performance using all three algorithms, this provides firm support for its stability and hence its suitability as a reference gene.

We have previously used these three algorithms to identify suitable reference genes in healthy and dystrophic murine ( Hildyard *et al.*, 2019) and canine muscle ( Hildyard *et al.*, 2018). Here we focus on the brain, identifying reference genes suitable for normalising gene expression in both healthy and dystrophic dogs and mice. We prepared a comprehensive sample set of healthy and dystrophin-deficient *mdx* mouse brain tissues (N=5 per genotype), divided into three brain regions (cortex, cerebellum, brainstem), as well as healthy and dystrophin-deficient *DE50-MD* dog brain tissues (N=5 per genotype), divided into six brain regions (olfactory bulbs, frontal cortex, temporal cortex, occipital cortex, brainstem, cerebellum). We used two complementary panels of species-specific reference genes (with shared orthologs where possible): nine candidate reference genes for canine brain samples, and eleven for mouse ( Table 1). These panels included those reported as suitable reference genes in the brains of humans ( *GAPDH, SHDA, UBC, B2M, CYC1*)( Coulson *et al.*, 2008; Penna *et al.*, 2011), rodents ( *YWHAZ, RPL13a, UBC, HRPT, 18S, B2M*)( Boda *et al.*, 2009; Bonefeld *et al.*, 2008; Kang *et al.*, 2018; Ramhoj *et al.*, 2019) and pigs ( *ACTB, YWHAZ*), three widely-used ‘housekeeping genes’ ( *18S*, *GAPDH* and *ACTB*)( Abe *et al.*, 2009; Baker *et al.*, 2006; Camerino *et al.*, 2014; DiMario, 2018; Feder *et al.*, 2014; Wright *et al.*, 2017, and those that performed well in muscle samples in dystrophic dogs ( *HPRT1*, *SDHA*, *RPL13a*)( Hildyard *et al.*, 2018). Using this panel, we identified suitable reference genes for normalisation of gene expression in healthy and dystrophic canine and murine brain tissue.

**Table 1.** Candidate reference genes assessed in murine and canine normal and dystrophic brain samples.

Canine Reference Genes	Murine Reference Genes
*HPRT1*	*HPRT1*
*RPL13a*	*RPL13a*
*SDHA*	*SDHA*
*18S*	*18S*
*ACTB*	*ACTB*
*B2M*	*B2M*
*UBC*	*UBC*
*GAPDH*	*GAPDH*
*YWHAZ*	*ZFP91*
	*MON2*
	*CYC1*

## Methods

### Ethical considerations

This work was conducted under UK Home Office Project Licences numbers P9A1D1D6E (dog) and PPL 70–7777 (mice) and was approved by the Royal Veterinary College Ethics and Welfare committee.

### Animals

A total of 10 mice and 10 dogs were used in this study (experimental unit: individual animal). Sample size was determined by available WT dog brain tissue. Brain tissue was collected post-mortem from 5 dystrophic DE50-MD male dogs and 5 age-matched wildtype (WT) control male dogs (median age 15 months, range 6–19), as part of a natural history study conducted under UK Home Office Project Licence. Dogs were housed in a dedicated canine facility with large indoor pens alongside access to outdoor runs and grass paddocks. Carrier female Beagle (RCC strain)-cross (F3 generation) dogs derived from an original founder Bichon-Frise cross Cavalier King Charles Spaniel female carrier were mated with male Beagles (RCC strain) to produce DE50-MD, carrier and wildtype offspring. Adult dogs were group housed (12 hour light/dark cycle; 15–24°C) until females were close to whelping; thereafter, pregnant females (singly housed) were allowed to whelp naturally and all puppies within a litter (including those on trials) were kept with their mother in a large pen, to enable nursing with access to a bed under a heat lamp (~28°C). From 4 weeks of age, puppies also received puppy feed (Burns, *ad lib*) until weaning at 12 weeks. Dogs over the age of 12 weeks received 2 feeds daily and *ad lib* water. All animals follow a comprehensive socialisation programme and twice daily welfare assessments.

Brain samples were obtained post-mortem from 5 male *mdx* mice and 5 healthy strain and age-matched male C57BL/10 WT mice (median age 7 months, range 6–8), bred under UK Home Office Project Licence and approved by the Royal Veterinary College Ethics and Welfare committee. Mice were housed in open top cages in a minimal disease unit at an average 21°C in a 12 hours light/ 12 hours dark light cycle with food and water provided *ad lib*. 

### Tissue collection

Dogs were euthanised by pentobarbital intravenous injection and mice by cervical dislocation. All efforts were made to ameliorate any suffering of animals with euthanasia performed by a highly experienced scientist (DW or RP). The brain was collected and dissected (delay between euthanasia and brain collection: <10 mins for mice, <2 hours for dogs). For canine brains, approximately 1–2 cm ^3^ samples of regions of interest (olfactory bulbs, frontal cortex, temporal cortex, occipital cortex, brainstem, cerebellum) were snap frozen in liquid nitrogen. Whole mouse brains were dissected into cortex, cerebellum and brainstem and snap frozen as above. No animals were excluded from analysis.

### RNA isolation and cDNA synthesis

Frozen tissues were pulverised under liquid nitrogen using a mortar and pestle and 50–100 mg of the powder was placed directly into 1ml of TRIzol reagent (Invitrogen). RNA was extracted following the manufacturer’s instructions (with inclusion of an additional 1:1 chloroform extraction following phase separation, and inclusion of 10μg glycogen during precipitation to maximise RNA yield, as previously described)( Hildyard *et al.*, 2019; Hildyard *et al.*, 2018). RNA purity was assessed by spectrometry (Nanodrop ND1000). Samples with guanidium carryover (260/230 <1.7) underwent a second isopropanol precipitation step.

The RTnanoscript2 kit (PrimerDesign) was used to prepare cDNA from 1.6μg of total RNA per 20μl reaction. Both random 9mer and oligodT priming was used. cDNA samples were then diluted (1/20) to give a final cDNA concentration of approximately 4 ng/μl (assuming 1:1 conversion of RNA to cDNA).

### qPCR and analysis

qPCR reactions were performed in 10μl volumes in triplicate with 2μl cDNA per well (approx 8ng), using PrecisionPLUS SYBR green mastermix (PrimerDesign) with primers to *ActB*, *UBC*, *18S*, *SDHA*, *RPL13a*, *YWHAZ2*, *B2M* ( Table 1) taken from the geNorm *Canis familiaris* set (PrimerDesign), and primers to *MON2*, *ZFP91*, *HTATSF1*, *GAPDH*, *ACTB*, *18S*, *SDHA*, *RPL13a*, *PAK1IP1* and *B2M* taken from the geNorm and geNorm- PLUS *Mus musculus* primer sets (Primerdesign). All sequences are proprietary property of Primerdesign, however context sequence and anchor nucleotides have been published previously ( Hildyard & Wells, 2014; Hildyard *et al.*, 2019). Primers to *HPRT1* were the pan-species set taken from Valadan *et al.* (2015) and have the following sequence:

HPSF F 5’-GGACTAATTATGGACAGGACTG-3’

HPSF R 5’-GCTCTTCAGTCTGATAAAATCTAC-3’

Primers were designed to the ensemble canine GAPDH sequence (ENSCAFT00000023939) using primer3 (primer3.ut.ee) and have the following sequence:

F 5’-TGAATGTCGGAGTGAACGGA-3’

R 5’-TGACTGTGCCGTGGAATTTG-3’

GAPDH primers were designed to span intronic sequence to minimise amplification of genomic DNA (mammalian genomes contain multiple GAPDH pseudogenes) ( Sun *et al.*, 2012). 

PCR was conducted in a CFX384 light cycler (BioRad) using a three-step reaction (95 °C, 15sec; 60 °C, 20sec; 72 °C, 20sec for 40 cycles) with subsequent melt curves performed for all reactions. All primer pairs gave sharp, single amplicon products and single melt peaks. Quantification cycle (Cq) values were determined by regression.

Candidate reference genes were analysed using geNorm (Version 3), NormFinder and Bestkeeper (Version 1), using the Windows 7 operating system and Excel 2003 or 2010 (Microsoft). Raw Cq values were used for Bestkeeper analysis, while each gene was linearized by conversion to relative quantities (RQ) for geNorm and NormFinder analysis. 

Dog and mouse data were analysed separately, with datasets in the following groups:

- Entire dataset (all samples, all brain regions)
- Healthy samples (all brain regions, non-dystrophic only)
- Dystrophic samples (all brain regions, dystrophic only)
- Olfactory bulb samples (dystrophic and non-dystrophic) (canine samples only)
- Cortical samples (dystrophic and non-dystrophic)
- Cerebellar samples (dystrophic and non-dystrophic)
- Brainstem samples (dystrophic and non-dystrophic)

For Normfinder analysis, datasets were assessed either as *ungrouped* (to determine overall expression variation as with geNorm and Bestkeeper), or *grouped* (to determine variation in expression over datasets between specific, user-specified groups) as shown:

- Individual animal (10 dogs, 10 mice)
- Dystrophic/non-dystrophic (2 groups)
- Brain region (6 in dogs, 3 in mice)

## Results

### Cq determinations

**Figure 2.**
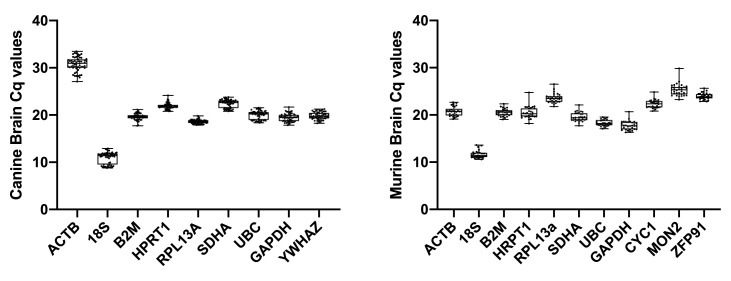
Raw Cq values for each of the assessed genes in canine brain ( **A**) and murine brain ( **B**). N=10 per gene per species.

For each assessed gene, individual Cq values were relatively consistent ( Figure 2, Extended data Tables 1, 2 ( Crawford *et al.*, 2021c)). *18S* showed the highest level of expression, while *ACTB* and *MON2* showed the lowest in dog and mouse, respectively. 

### Canine brain

***geNorm analysis.*** The geNorm algorithm uses a pairwise approach to rank genes by their average expression stability (M), with lower scores representing higher stability. Sequential removal of the gene demonstrating the least correlation (the highest M value), followed by recalculation of M values, results in the eventual identification of a single pair of highly correlated genes, considered the ‘best pair’.

**Figure 3.**
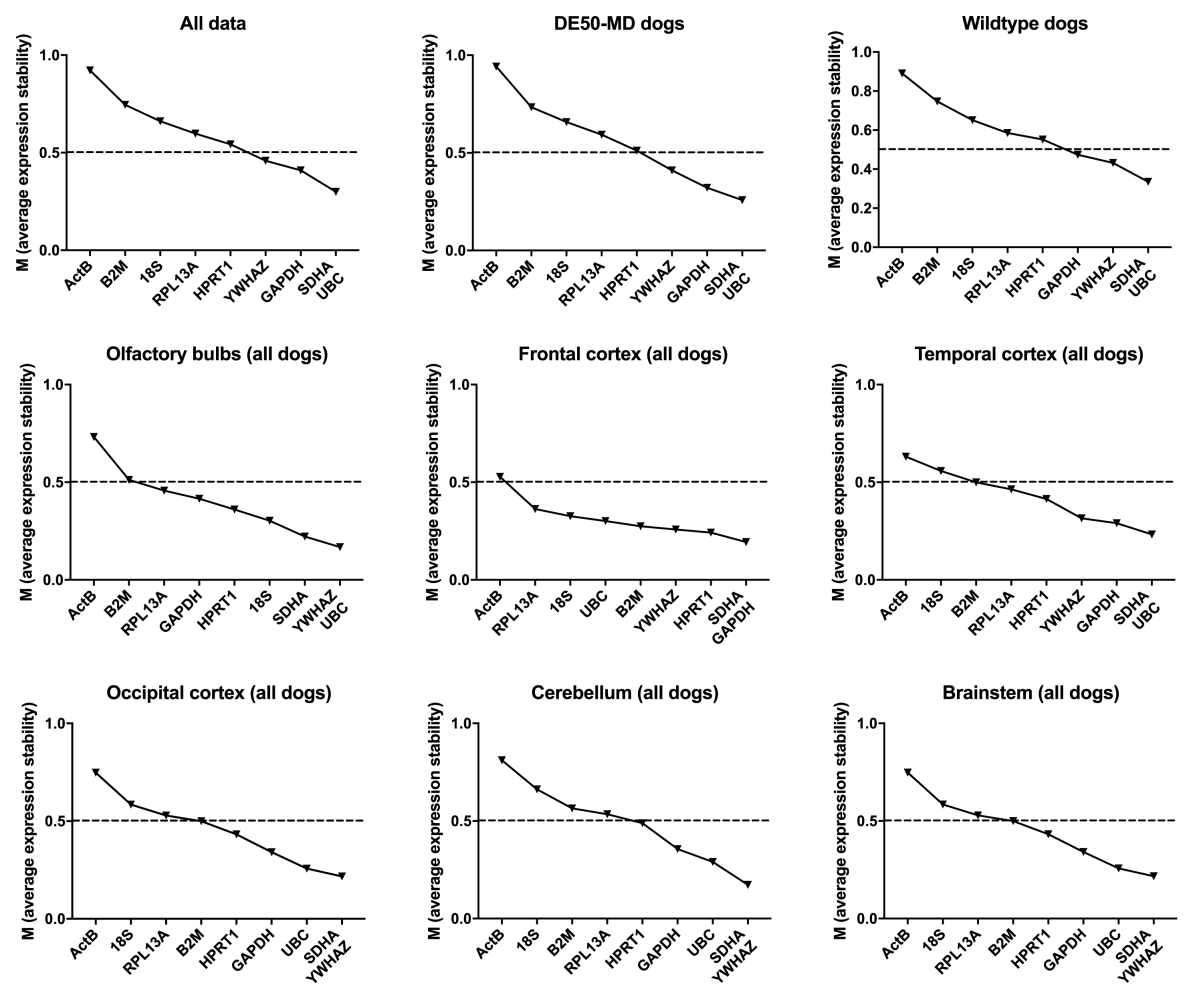
geNorm analysis for all canine dataset groups. The average expression stability M is shown for the full dataset or specified subgroups (ranking left to right: least stable to most stable). Dashed line: M = 0.5 (reported threshold of stability for strong candidates).

Analysis of our entire dataset ( Crawford *et al.*, 2021a), or segregated by genotype (WT or DE50-MD) revealed consistency both in overall score and in ranking, suggesting that healthy and dystrophic brains do not exhibit marked transcriptional changes ( Table 2, Figure 3). The commonly accepted geNorm threshold for suitable reference gene stability is M < 0.5 (bold gene names in Table 2). *SDHA* and *UBC* formed the best pair in all three comparisons, with *YWHAZ* and *GAPDH* ranking third or fourth, while *ACTB* and *B2M* were the lowest scoring genes.

**Table 2.** geNorm output for the full canine dataset or specified subgroups. Genes are scored from highest ranking pair (top) to least stable gene (bottom). Bold: score < 0.5 (reported threshold for suitability); italics: score > 0.75 (considered poor reference genes).

	All samples	DE50-MD dogs	Wildtype dogs	Olfactory bulb	Frontal cortex	Temporal cortex	Occipital cortex	All cortical regions	Cerebell- um	Brainstem
**Most ** **stable** **(Best Pair)**	**SDHA ** **UBC**	**SDHA** **UBC**	**SDHA UBC**	**YWHAZ** **UBC**	**SDHA ** **GAPDH**	**SDHA UBC**	**SDHA** **YWHAZ**	**B2M** **HPRT1**	**SDHA ** **YWHAZ**	**SDHA** ** YWHAZ**
	**GAPDH**	**GAPDH**	**YWHAZ**	**SDHA**	**HRPT1**	**GAPDH**	**UBC**	**RPL13A**	**UBC**	**UBC**
	**YWHAZ**	**YWHAZ**	**GAPDH**	**18S**	**YWHAZ**	**YWHAZ**	**GAPDH**	**YWHAZ**	**GAPDH**	**GAPDH**
	HPRT1	HPRT1	HPRT1	**HPRT1**	**B2M**	**HPRT1**	**HPRT1**	**UBC**	**HPRT1**	**HPRT1**
	RPL13A	RPL13A	RPL13A	**GAPDH**	**UBC**	**RPL13A**	**B2M**	SDHA	RPL13A	**B2M**
	18S	18S	18S	**RPL13A**	**18S**	B2M	RPL13A	18S	B2M	RPL13A
	*B2M*	B2M	B2M	B2M	**RPL13A**	18S	18S	GAPDH	18S	18S
**Least** **stable**	*ACTB*	*ACTB*	*ACTB*	ACTB	ACTB	ACTB	ACTB	ACTB	*ACTB*	ACTB

geNorm analysis by brain region again ranked *ACTB* lowest by all analysed subsets, with *YWHAZ, SDHA* and *UBC* highly ranked. The best pair varied by brain region, as did overall stabilities, although all subsets exhibited greater overall stability (lower M) than the combined dataset, as might be expected for more homogenous tissues. Greater diversity was found between cortical regions, with frontal and occipital cortex scoring *HPRT1* and (unexpectedly) *B2M* highly in these datasets, which also formed the best pair when all cortical samples were combined. However, between 6 and 8 of the assessed genes scored < 0.5 in the assessed cortical regions, suggesting that these regions might exhibit generally high transcriptional stability.

Calculation of the pairwise variation resulting from inclusion of additional reference genes was also undertaken using the geNorm algorithm. This provides an indication of whether the addition of further genes will improve normalisation. While increasing the number of reference genes tended to lower the overall variation (Extended data Table 3 ( Crawford *et al.*, 2021c)), values below 0.2 are considered acceptable ( Vandesompele *et al.*, 2002b) and the suggested “best” pair of genes alone was consistently sufficient to pass this threshold.

**Figure 4.**
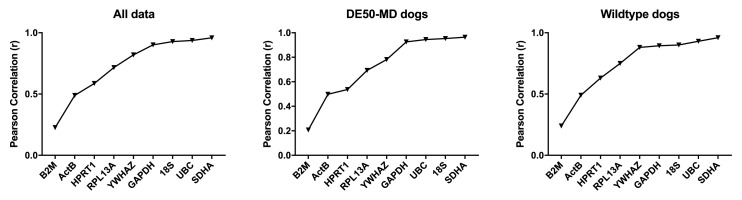
BestKeeper analysis of canine data. Correlation coefficients for the assessed genes are shown for the full dataset, or DE50-MD or WT dogs alone, ranked from least to most stable (left to right).

***BestKeeper analysis.*** This algorithm effectively determines which gene best reflects the behaviour of the dataset as a whole. It uses a pairwise comparison of each gene to the geometric average of all assessed genes (the ‘bestkeeper’), ( Pfaffl *et al.*, 2004b). The Pearson correlation coefficient (r) is used to rank genes and r = 1 indicates perfect correlation with the bestkeeper. *SDHA, UBC, YWHAZ* and *GAPDH* consistently scored highly, while *ACTB* and *B2M* demonstrated low correlation values in the canine brain, though here the latter was markedly lower scoring than the former ( Table 3, Figure 4). In contrast to the geNorm analysis, *18S* represented one of the highest scoring candidates, with r > 0.85 in 7/9 of the candidate reference genes, and the trio of *UBC, SDHA* and *18S* were similar in score. Analysis of brains by region largely accorded with this: in most comparisons, *UBC, SDHA, YWHAZ, GAPDH* and *18S* ranked highly, while *ACTB* and *B2M* occupied the lowest ranks. *HPRT1*, in contrast, scored poorly overall, yet was ranked highly in olfactory bulbs and occipital cortex specifically, and indeed performed acceptably in temporal cortex and brainstem. This interesting pattern is best explained by modest but consistent region-specific variation in expression. A gene that shows consistent good correlation with the Bestkeeper across the entire sample set will score highly overall, but a gene with excellent within-region correlations that nevertheless varies between brain regions will appear more biphasic in correlation, scoring less highly overall (Extended data Figure 1 ( Crawford *et al.*, 2021c)). 

**Table 3.** Bestkeeper output for the full canine dataset or specified subgroups. Genes are ranked from top to bottom by Pearson correlation (r) with the BestKeeper. Bold: r > = 0.85; italics: r < 0.6.

	All samples	DE50-MD dogs	Wildtype dogs	Olfactory bulb	Frontal cortex	Temporal cortex	Occipital cortex	Cerebell- um	Brainst- em
**Most** **stable**	**SDHA**	**SDHA**	**SDHA**	**YWHAZ**	**SDHA**	**18S**	**SDHA**	**YWHAZ**	**UBC**
	**UBC**	**18S**	**UBC**	**HRPT1**	**GAPDH**	**SDHA**	**HRPT1**	**SDHA**	**GAPDH**
	**18S**	**UBC**	**18S**	**SDHA**	**18S**	**UBC**	**RPL13A**	**18S**	**YWHAZ**
	**GAPDH**	**GAPDH**	**GAPDH**	18S	**UBC**	**GAPDH**	**GAPDH**	**GAPDH**	**18S**
	YWHAZ	YWHAZ	**YWHAZ**	GAPDH	HRPT1	**YWHAZ**	**18S**	**UBC**	**SDHA**
	RPL13A	RPL13A	RPL13A	UBC	B2M	**HRPT1**	**UBC**	RPL13A	**HRPT1**
	*HRPT1*	*HRPT1*	*HRPT1*	RPL13A	RPL13A	**RPL13A**	**ACTB**	HPRT1	RPL13A
	*ACTB*	*ACTB*	*ACTB*	*B2M*	*YWHAZ*	ACTB	*YWHAZ*	*B2M*	B2M
**Least** **stable**	*B2M*	*B2M*	*B2M*	*ACTB*	*ACTB*	*B2M*	*B2M*	*ACTB*	*ACTB*

**Figure 5.**
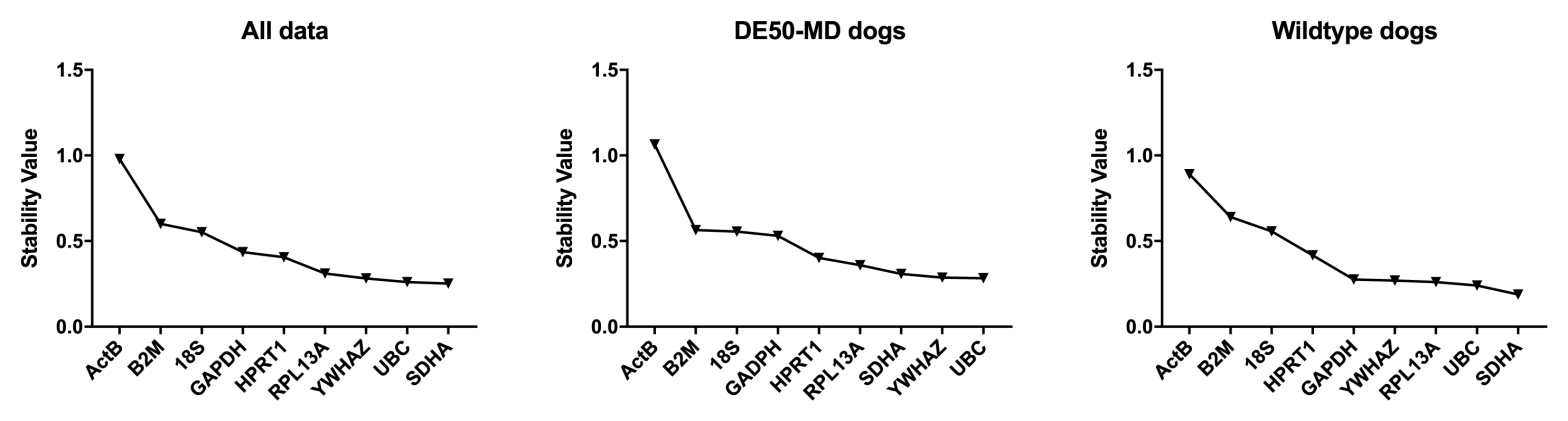
Normfinder analysis of canine samples (ungrouped). Stability values (left to right: least stable to most stable) for the reference gene candidates are shown for the entire dataset, DE50-MD and WT samples (as indicated).

***Normfinder analysis.*** The Normfinder algorithm differs from the pairwise approaches used by geNorm and Bestkeeper by evaluating each gene individually to determine absolute expression stability within the whole dataset or within specified subgroups ( Andersen *et al.*, 2004a). As shown ( Table 4 & Table 5, Figure 5) even under this alternative assessment methodology, *SDHA, UBC* and *YWHAZ* were highly ranked in the canine samples. *18S* performed poorly (in agreement with geNorm, but not Bestkeeper), while *RPL13A* joined the top-scoring candidates. Again, *HPRT1* was ranked modestly for the combined dataset, while scoring highly in brain regions individually (this gene was the highest ranked in olfactory bulbs, occipital cortex and brainstem). *GAPDH* ranked low in the combined scores, but interestingly was the most stable gene in the cerebellum and temporal cortex, potentially suggesting regional differences in its expression levels and respective stability. In agreement with both previous algorithms, *B2M* and *ACTB* were assigned poor stability values. 

**Table 4.** Normfinder output for the full ungrouped canine dataset or specified subgroups. Genes are ranked (top to bottom) from highest to lowest scoring. Bold: stability <0.25; italics: stability > 0.4.

	All samples	DE50-MD dogs	Wildtype dogs	Olfactory bulb	Frontal cortex	Temporal cortex	Occipital cortex	Cerebell- um	Brainstem
**Most** **stable**	SDHA	UBC	**SDHA**	**HPRT1**	**SDHA**	**GAPDH**	**HPRT1**	**GAPDH**	**HPRT1**
	UBC	YWHAZ	**UBC**	**RPL13A**	**GAPDH**	**UBC**	**SDHA**	**SDHA**	**UBC**
	YWHAZ	SDHA	RPL13A	**YWHAZ**	**HPRT1**	**YWHAZ**	**RPL13A**	YWHAZ	**YWHAZ**
	RPL13A	RPL13A	YWHAZ	UBC	**18S**	**SDHA**	**18S**	UBC	**SDHA**
	*HRPT1*	*HRPT1*	GAPDH	SDHA	**B2M**	**HPRT1**	**YWHAZ**	RPL13A	GAPDH
	*GAPDH*	*GAPDH*	*HRPT1*	18S	**YWHAZ**	RPL13A	**UBC**	HPRT1	RPL13A
	*18S*	*18S*	*18S*	*B2M*	**RPL13A**	B2M	**B2M**	*B2M*	B2M
	*ACTB*	*B2M*	*B2M*	*GAPDH*	**UBC**	*18S*	*GAPDH*	*18S*	*18S*
**Least** **stable**	*B2M*	*ACTB*	*ACTB*	*ACTB*	*ACTB*	*ACTB*	*ACTB*	*ACTB*	*ACTB*

**Table 5.** Normfinder output for the full canine dataset or specified subgroups. Genes are ranked from the highest to lowest scoring. Grouped analysis also indicates the best pair of genes for normalisation. (Bold: stability <0.25; italics: stability > 0.4).

	All data grouped by genotype	All data, grouped by dog	All data, grouped by brain region	All DE50-MD dog data grouped by dog	All DE50-MD dog data grouped by brain region	All WT data grouped by dog	All WT data grouped by brain region
**Best** ** pair**	**UBC and SDHA**	**RPL13A and ** **SDHA**	**RPL13A and** ** SDHA**	**RPL13A and UBC**	**RPL13A and SDHA**	**RPL13A and ** **SDHA**	**RPL13A and ** **SDHA**
**Most** **stable**	**SDHA**	**SDHA**	**SDHA**	**YWHAZ**	SDHA	**GAPDH**	**SDHA**
	**UBC**	**GAPDH**	**RPL13A**	**SDHA**	YWHAZ	**UBC**	**RPL13A**
	**YWHAZ**	**YWHAZ**	**UBC**	UBC	UBC	**SDHA**	UBC
	**RPL13A**	**UBC**	YWHAZ	HPRT1	GADPH	**RPL13A**	YWHAZ
	**GAPDH**	HPRT1	GAPDH	GADPH	RPL13A	**YWHAZ**	GAPDH
	**HPRT1**	RPL13A	HPRT1	RPL13A	*HPRT1*	**HPRT1**	*HPRT1*
	**18S**	B2M	*18S*	B2M	*B2M*	B2M	*18S*
	**B2M**	18S	*B2M*	18S	*18S*	18S	*B2M*
**Least** **stable**	**ACTB**	*ACTB*	*ActB*	*ActB*	*ActB*	*ActB*	*ActB*

Under grouped analysis ( Table 5) a similar pattern was observed, with *SDHA, UBC* and *YWHAZ* ranked highly, and *ACTB, 18S* and *B2M* rated as low stability candidates. Grouped analysis also suggests a ‘best pair’. Interestingly, *RPL13A* featured in the “best pair” for six of the studied groups, often alongside *SDHA*.

Grouping the entire dataset by brain region produced rankings comparable to ungrouped analysis, implying that any region-specific behaviours are sufficiently marked so as to be detectable without recourse to grouping. Grouping by genotype (WT/DE50-MD) or by individual animal produced rankings similar (if not identical) to ungrouped analysis, the former comparison indicating that no candidate genes within our dataset show clear disease-association (as also suggested by geNorm and Bestkeeper).

**Figure 6.**
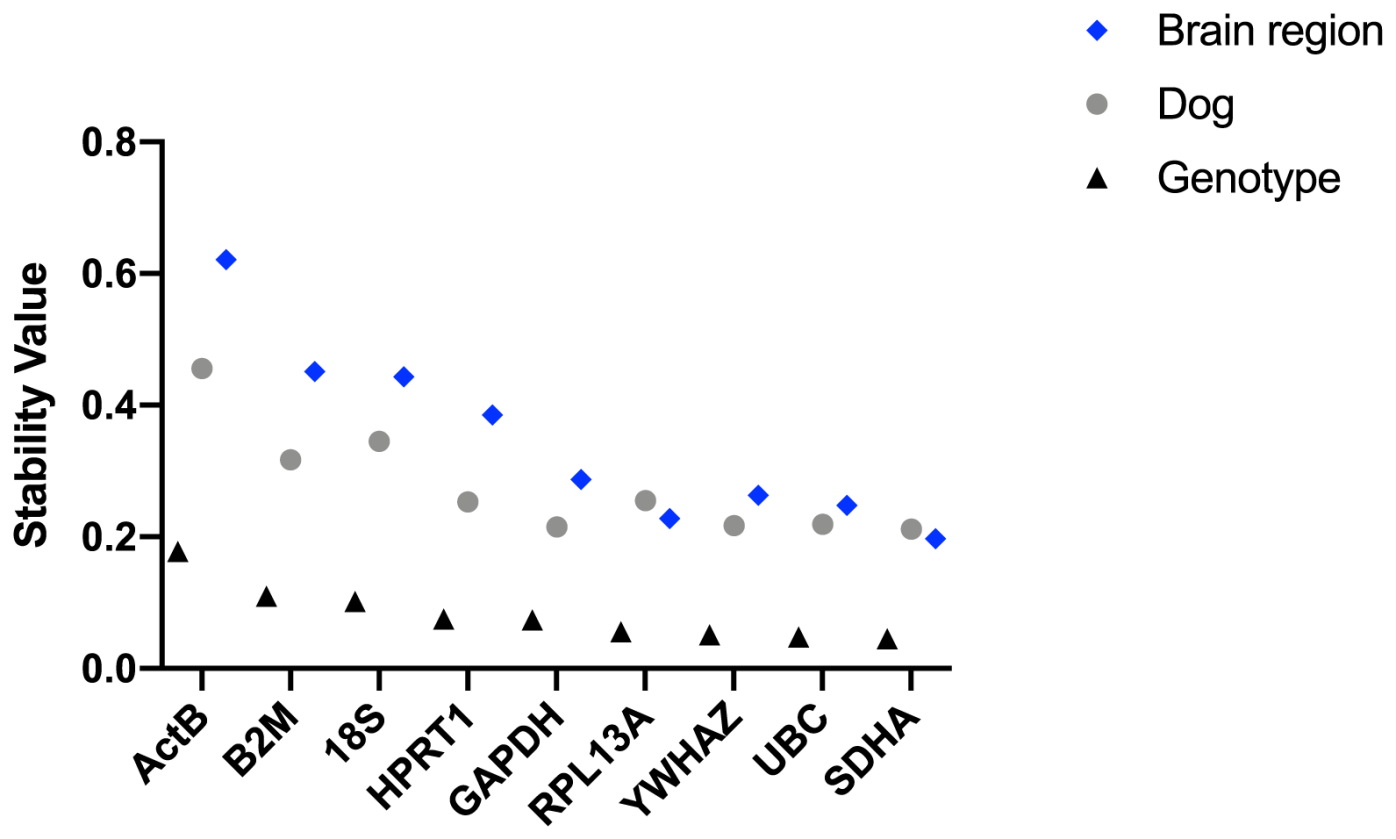
Normfinder analysis performed by grouping samples by genotype, by dog or by brain region. The stability value was lower (indicating higher stability) when grouped by genotype, compared to when grouped by individual dog.

Interestingly, when dogs were grouped by genotype, the stability values for all assessed genes were consistently lower (indicating higher stability) than when grouped by individual dog or by brain region ( Figure 6). This implies that genotype, with its associated deficiency of dystrophin in the brain, accounts for less variation in gene expression than does brain region or individual.

**Figure 7.**
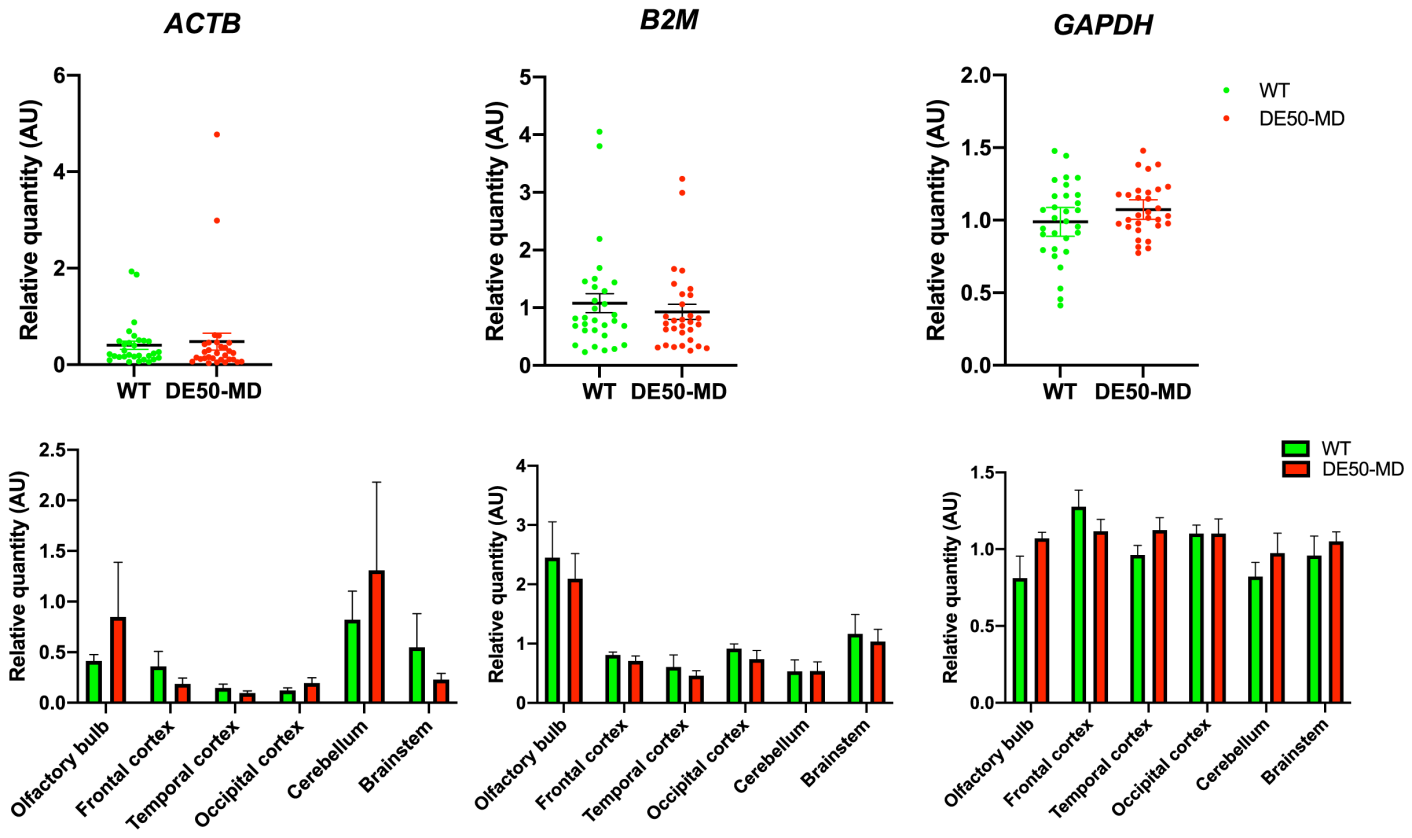
*ACTB* and *B2M* expression normalised to the geometric mean of *SDHA, UBC* and *YWHAZ.* Normalisation of *ACTB* proved inconclusive with no disease association and variable expression across the studied brain regions. Normalisation of *B2M* revealed elevated expression within the olfactory bulbs. Normalisation of GAPDH showed consistent expression across all studied brain regions and both genotypes.

***Summary and validation.*** *SDHA, UBC* and *YWHAZ* are the strongest overall candidates and are suitable for normalising gene expression in healthy and dystrophic canine brain tissue, regardless of the brain region studied. To attempt to validate these candidates and ascertain if low-ranking genes might show disease association, we employed a within-dataset strategy: using the geometric mean of the highest scoring genes to normalise those that consistently ranked poorly ( Figure 7). Normalisation of *ACTB* proved inconclusive with highly variable expression across regions and genotypes. Normalisation of *B2M* was more informative: while expression in most brain regions was comparable, this gene shows clear and consistent elevated expression within the olfactory bulbs. In comparison, normalisation of GAPDH (a higher scoring gene) revealed stable expression across all studied regions, in agreement with its higher ranking.

### Murine brain

**Figure 8.**
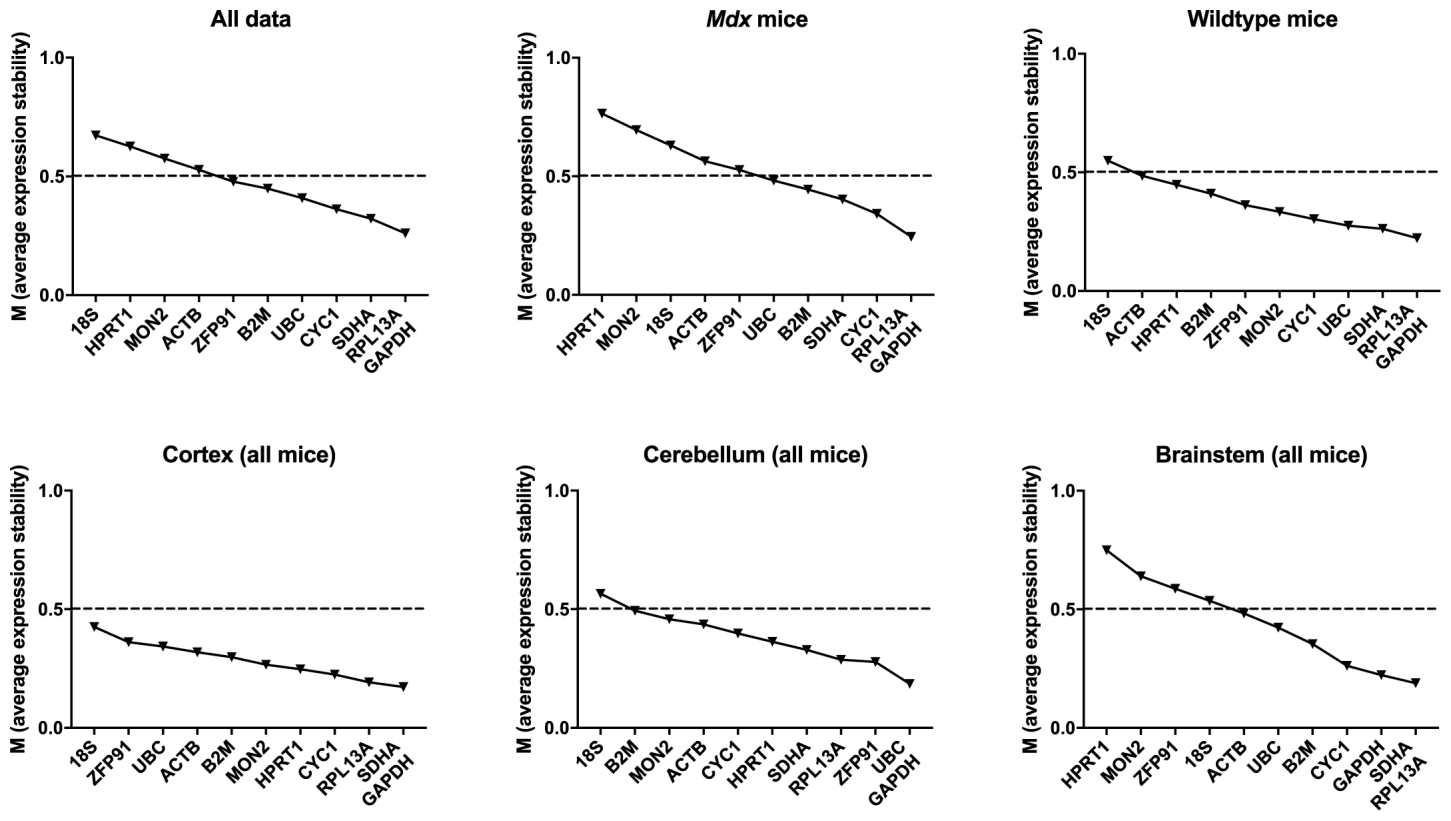
geNorm analysis for all murine dataset combinations. geNorm output ranked by average expression stability M (least stable to most stable are shown from left to right) for the full dataset, or specific subgroups (as indicated). Dashed line: M = 0.5 (reported threshold of stability for strong candidates).

***geNorm analysis.*** geNorm analysis revealed *RPL13A*, *GAPDH*, *SDHA* and *CYC1* as the highest ranking genes, but with 7 to 10 genes per grouping achieving M <0.5, it appears (as with canine brains) that individual brain regions exhibit high overall transcriptional stability ( Table 6, Figure 8) ( Crawford *et al.*, 2021b). Lowest ranking genes showed some variation across regions and genotypes, but most commonly consisted of *18S* and *HRPT1*.

**Table 6.** geNorm output for the full murine dataset or specified subgroups. Genes are ranked from the highest scoring pair (top) to the least stable gene (bottom). Bold: score < 0.5 (threshold for suitability); italics: score > 0.75 (poor candidate reference genes).

	All samples	*Mdx* mice	WT mice	Cortex	Cerebellum	Brainstem
**Most stable** ** (Best Pair)**	**RPL13A** ** GAPDH**	**RPL13A** ** GAPDH**	**RPL13A** ** GAPDH**	**SDHA GAPDH**	**UBC GAPDH**	**SDHA RPL13A**
	**SDHA**	**CYC1**	**SDHA**	**RPL13A**	**ZFP91**	**GADPH**
	**CYC1**	**SDHA**	**UBC**	**CYC1**	**RPL13A**	**CYC1**
	**UBC**	**B2M**	**CYC1**	**HPRT1**	**SDHA**	**B2M**
	**B2M**	**UBC**	**MON2**	**MON2**	**HPRT1**	**UBC**
	**ZFP91**	ZFP91	**ZFP91**	**B2M**	**CYC1**	**ACTB8**
	ACTB	ACTB	**B2M**	**B2M**	**ACTB**	18S
	MON2	18S	**HPRT1**	**UBC**	**MON2**	ZFP91
	HPRT1	MON2	**ACTB**	**ZFP91**	**B2M**	MON2
**Least stable**	18S	*HPRT1*	18S	**18S**	18S	*HPRT1*

**Figure 9.**
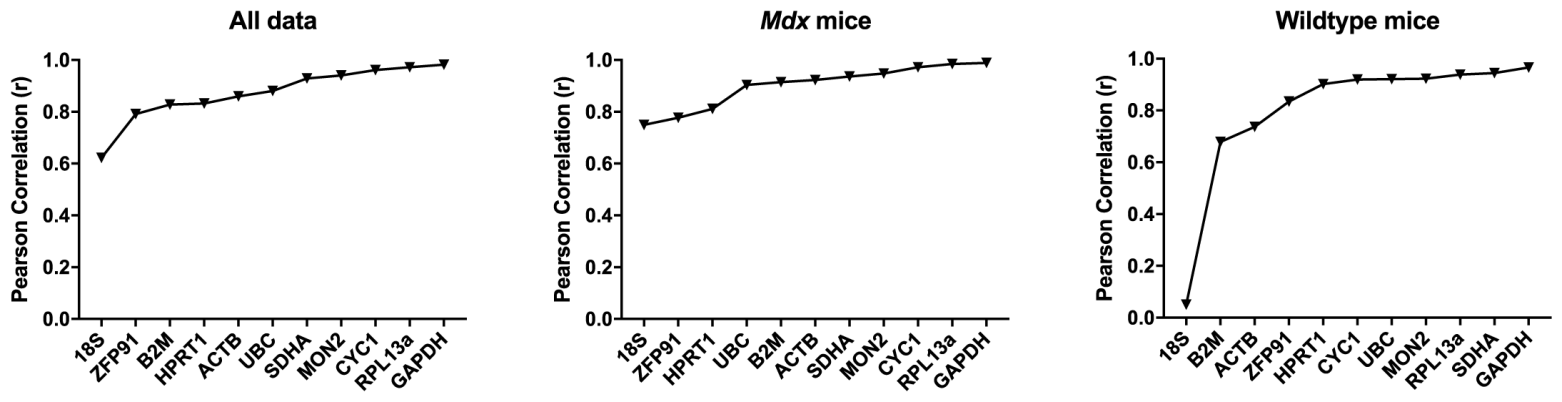
BestKeeper analysis for murine data. Correlation coefficients for the assessed gene candidates are shown for the full dataset, or *mdx* or WT mice alone (as indicated), ranked from least to most stable (left to right).

As for the canine brain, calculation of the pairwise variation resulting from inclusion of additional reference genes revealed that increasing the number of reference genes to 3, 4 or 5 tended to lower the overall variation (Extended data Table 4 ( Crawford *et al.*, 2021c)), however the use of the suggested “best” pair of genes was consistently sufficient to exceed the acceptable threshold of 0.2 ( Vandesompele *et al.*, 2002b),

***Bestkeeper Analysis.*** In the murine brain, the majority of the assessed genes were high scoring, with r > 0.85. *GAPDH, CYC1* and *RPL13A* were consistently high scoring, while *18S, B2M* and *ZFP91* scoring were the lowest scoring ( Table 7, Figure 9).

**Table 7.** Bestkeeper output for the full murine dataset or specified subgroups. Genes are ranked from top to bottom by Pearson correlation (r) with the BestKeeper. Bold: r > = 0.85; italics: r < 0.6.

	All samples	*Mdx* mice	WT mice	Cortex	Cerebellum	Brainstem
**Most stable**	**GAPDH**	**GAPDH**	**GAPDH**	**GAPDH**	**MON2**	**GAPDH**
	**RPL13a**	**RPL13a**	**SDHA**	**CYC1**	**GAPDH**	**MON2**
	**CYC1**	**CYC1**	**RPL13a**	**HPRT1**	**ACTB**	**RPL13a**
	**MON2**	**MON2**	**MON2**	**MON2**	**CYC1**	**CYC1**
	**SDHA**	**SDHA**	**UBC**	**RPL13a**	**RPL13a**	**SDHA**
	**UBC**	**ACTB**	**CYC1**	**SDHA**	**UBC**	**UBC**
	**ACTB**	**B2M**	**HPRT1**	**B2M**	**ZFP91**	**B2M**
	HPRT1	**UBC**	ZFP91	**ACTB**	SDHA	**ACTB**
	B2M	HPRT1	ACTB	**ZFP91**	HPRT1	HPRT1
	ZFP91	ZFP91	B2M	**UBC**	*18S*	18S
**Least stable**	18S	18S	*18S*	*18S*	*B2M*	ZFP91

**Figure 10.**
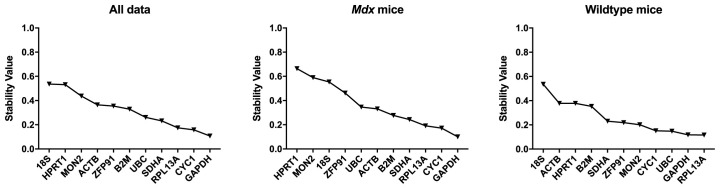
Normfinder analysis of murine samples (ungrouped). Stability values for the assessed gene candidates are shown for the full dataset, *mdx* mice and WT mice (as indicated) (left to right: least to most stable).

***Normfinder Analysis.*** Normfinder results were in agreement with that of the Bestkeeper analysis, with *GAPDH, CYC1* and *RPL13A* consistently highest ranking ( Table 8, Figure 10). *18S, HPRT1* and *ZFP91* were lowest ranking.

**Table 8.** Normfinder output for the full ungrouped murine dataset or specified subgroups. Genes are ranked (from highest to lowest scoring (top to bottom). Bold: stability <0.25; italics: stability > 0.4.

	All samples	*Mdx* mice	Control mice	Cortex	Cerebellum	Brainstem
**Most stable**	**GAPDH**	**GAPDH**	**RPL13A**	**CYC1**	**GAPDH**	**GAPDH**
	**CYC1**	**CYC1**	**GAPDH**	**HPRT1**	**UBC**	**CYC1**
	**RPL13A**	**RPL13A**	**UBC**	**SDHA**	**CYC1**	**SDHA**
	**SDHA**	**SDHA**	**CYC1**	**GAPDH**	**ZFP91**	**RPL13A**
	UBC	B2M	**MON2**	**B2M**	**RPL13A**	B2M
	B2M	ACTB	**ZFP91**	**RPL13A**	SDHA	UBC
	ZFP91	UBC	**SDHA**	**ACTB**	ACTB	ACTB
	ACTB	*ZFP91*	B2M	**MON2**	MON2	*18S*
	*MON2*	*18S*	ACTB	**UBC**	HPRT1	*MON2*
	*HPRT1*	*MON2*	HPRT1	ZFP91	B2M	*ZFP91*
**Least stable**	*18S*	*HPRT1*	*18S*	*18S*	*18S*	*HPRT1*

Under grouped analysis ( Table 9) a similar pattern was observed, with *GAPDH, CYC1* and *RPL13A* ranked highly, while *HPRT1, 18S* and *MON2* were universally rated as low stability candidates. Again, higher stability values were seen when samples were grouped by genotype, compared to when grouped by individual mouse or brain region. As with the canine samples, *RPL13A* performed well in the Normfinder grouped analysis and featured in the “best pair” in 5 of the 7 subgroups studied.

**Table 9.** Normfinder output for the full murine dataset or specified subgroups. Genes are ranked from highest scoring (lowest stability value) to lowest scoring. Grouped analysis also suggests the best pair of genes for normalisation. (Bold: stability <0.25; italics: stability > 0.4).

	All data grouped by genotype	All data, grouped by mouse	All data, grouped by brain region	All *mdx* mice data grouped by mouse	All *mdx* mice data grouped by brain region	All WT data grouped by mouse	All WT data grouped by brain region
**Best** ** pair**	**RPL13A and** ** GAPDH**	**RPL13A and** ** GAPDH**	**GAPDH and** ** CYC1**	**RPL13A and ** **GAPDH**	**SDHA and GAPDH**	**RPL13A and** ** UBC**	**RPL13A and** ** GAPDH**
**Most** **stable**	**GAPDH**	**GAPDH**	**RPL13A**	**GAPDH**	**GAPDH**	**RPL13A**	**RPL13A**
	**RPL13A**	**RPL13A**	**GAPDH**	**RPL13A**	**CYC1**	**UBC**	**GAPDH**
	**CYC1**	**SDHA**	**CYC1**	**SDHA**	**RPL13A**	**GAPDH**	**UBC**
	**SDHA**	**CYC1**	**ZFP91**	**CYC1**	**SDHA**	**CYC1**	**CYC1**
	**UBC**	**UBC**	**SDHA**	**B2M**	**ACTB**	**ZFP91**	**MON2**
	**B2M**	**B2M**	**UBC**	**ZFP91**	**B2M**	**SDHA**	**ZFP91**
	**ZFP91**	**ZFP91**	**B2M**	**UBC**	**ZFP91**	**MON2**	**SDHA**
	**ACTB**	**ACTB**	HPRT1	**ACTB**	**UBC**	**B2M**	B2M
	**MON2**	**MON2**	ACTB	18S	HPRT1	**ACTB**	ACTB
	**HPRT1**	18S	MON2	MON2	18S	HPRT1	HPRT1
**Least** **stable**	**18S**	HPRT1	18S	HPRT1	MON2	18S	18S

In summary, all three algorithms support use of *GAPDH, RPL13A* and *CYC1* as suitable for normalising gene expression in healthy and dystrophic murine brain tissue, regardless of the brain region studied. *MON2, ZFP91, 18S* and *HPRT1* were consistently the lowest ranking candidate reference genes, suggesting they are not an appropriate choice.

As with canine brains (above), we used the geometric mean of our three top reference genes ( *GAPDH, RPL13A* and *CYC1)* to normalise three low scoring genes ( *MON2, ZFP91, 18S)* to ascertain if these latter genes show disease association. No significant difference was found between brain from normal and dystrophic mice using the respective normalisation factor, suggesting that the expression of these lower scoring genes is not significantly altered by dystrophin deficiency within the studied brain regions. However, each gene showed variation in expression between brain regions. In comparison, *UBC*, a higher scoring gene, showed consistent expression levels across the three studied brain regions (Extended data Figure 1 ( Crawford *et al.*, 2021c)).

## Discussion

Analysis of gene expression by RT-qPCR is a key component of the investigative toolkit in deciphering the function of dystrophin in the brain. However, the inherently dynamic behaviour of mRNA, alongside the inevitable variations in efficiency of its extraction and conversion to cDNA, necessitate the use of a panel of stably-expressed reference genes for normalising measured expression data ( Hildyard & Wells, 2014; Palombella *et al.*, 2017; Schmittgen & Zakrajsek, 2000). In this study we have identified reference genes suitable for use in brain tissue from two important animal models of Duchenne muscular dystrophy, the DE50-MD dog and the *mdx* mouse. *SDHA, UBC* and *YWHAZ* had stable expression in canine brain, and *GAPDH, RPL13A* and *CYC1* in murine brain *.* These genes score highly by all three utilised algorithms, both within the full dataset and when assessed as subgroups, supporting a high stability. Furthermore, the identified panels of reference genes remained valid between dystrophic and normal brain samples, as well as across brain regions.

A recent study reported *YWHAZ, UBC and SDHA* (alongside *HMBS*) to be a suitable reference panel for use in human brain from both normal control patients and patients with Alzheimer’s disease ( Coulson *et al.*, 2008). Furthermore, *YWHAZ* (14-3-3 protein zeta) was a suitable reference gene in the developing and injured mouse CNS ( Xu *et al.*, 2018), and *UBC* (ubiquitin C) in rat cerebral cortex ( Ramhoj *et al.*, 2019). *SDHA (*succinate dehydrogenase subunit A) is a mitochondrial enzyme component and is abundantly expressed in the mitochondria-rich brain. It has previously scored highly as a reference gene in mouse brain ( Cheung *et al.*, 2017) and also in canine skeletal muscle (normal and dystrophic), likely reflecting the high mitochondrial content of skeletal muscle. Interestingly, however, a recent study of human brain samples found SDHA to be expressed at a low level (Ct mean >35) and so it was excluded from further analysis ( Rydbirk *et al.*, 2016, emphasising the potential difference between specific experimental designs (including primer sequences), sample types and sample quality. 

Different transcripts can exhibit markedly different stabilities. Our murine samples were collected and frozen within 10 minutes of death, compared with 120 minutes for our canine samples, which may be sufficient time to elicit post-mortem alterations in expression patterns. We found *ACTB* (cytoskeletal beta isoform) to be expressed at very low levels in canine brain: a finding in contrast to mouse brain and reported values for human brains ( Rydbirk *et al.*, 2016). In dogs, this transcript might experience rapid turnover post-mortem (supported by similar findings in canine muscle), so accounting for its poor ranking ( Hildyard *et al.*, 2018). Furthermore, our murine samples were collected in batches of 2 and 3 animals, while canine samples were collected from each dog on a separate date (post-mortem examinations were conducted on a single individual dog per day). Such batch effect could influence sample quality. Interestingly, the grouped Normfinder analysis revealed that greater sample variability (higher stability values) arose when dogs were grouped by individual animal, compared to when grouped by genotype. This would suggest that the brain of the dystrophin-deficient dog is transcriptionally very similar to that of the WT dog with regards to the assessed housekeeping genes, and that post-mortem changes and specific sample handling/preservation between individual animals could account for relatively greater variability in transcript detection. Multiple factors might vary between individual animals including interval between euthanasia and sample collection, sample handling, and ambient temperature. Furthermore, individual genetic variation is greater in the dogs due to endeavours to maintain an outbred canine colony, compared with the inbred mouse colonies used in this study. Similar factors likely apply in assessment of other large mammals, including humans.

*GAPDH* (Glyceraldehyde 3-phosphate dehydrogenase) is a glycolytic enzyme frequently employed as a reference gene given its relatively constitutive expression across cell and tissue types ( Penna *et al.*, 2011). We found it to exhibit stable expression in the healthy and dystrophic murine brain, but this gene also scored comparatively highly in canine samples. In agreement with our results, *GAPDH* has stable expression in the human brain, particularly the cerebellum, in patients with various neurodegenerative diseases as well as in normal control patients ( Coulson *et al.*, 2008; Grunblatt *et al.*, 2004). Interestingly, *GAPDH* was a poor candidate for normalisation in murine muscle, showing active disease association and prominent muscle-specific expression patterns ( Hildyard *et al.*, 2019).

*RPL13a* codes for a protein component of the large ribosomal subunit and scored highly in canine and murine muscle ( Hildyard *et al.*, 2019; Hildyard *et al.*, 2018). Genes directly associated with translational machinery have been found to score highly as candidate reference genes under a variety of conditions ( Kaur *et al.*, 2018; Molina *et al.*, 2018; Nakayama *et al.*, 2018), implying that translational components are very stable. Interestingly, both *RPL13a* and *CYC1* (Cytochrome C1) were identified as suitable reference genes in a study of 98 brain samples from two regions (prefrontal cortex and cerebellum) from human patients with neurodegenerative diseases (Alzheimer's disease, Parkinson's disease, Multiple System Atrophy, and Progressive Supranuclear Palsy) ( Rydbirk *et al.*, 2016). *CYC1* encodes a protein that forms part of the mitochondrial electron transport chain and is crucial in cellular respiration ( Schagger *et al.*, 2004).

*B2M* has previously been shown to be upregulated by approximately 2-fold in canine dystrophic muscle ( Hildyard *et al.*, 2018). B2M is highly expressed in immune lineages and so the persistent inflammatory state characteristic of dystrophic muscle might feasibly increase B2M gene expression. We did not identify an upregulation of B2M in dystrophic brain tissue, which is in agreement with the lack of inflammation in brains from DMD patients ( Dubowitz & Crome, 1969; Jagadha & Becker, 1988; Rosman & Kakulas, 1966). None of the candidate reference genes assessed in this study showed a significant difference in expression between healthy and dystrophic samples, suggesting that disease-specific alterations in expression patterns are likely to be subtle. Further studies to evaluate a wider spectrum of candidate genes might reveal novel disease-associated genes and so provide insights into dystrophin’s role within the brain.

The highest-scoring reference genes identified did not overlap between murine and canine brain samples, as we also reported in murine and canine muscle ( Hildyard *et al.*, 2019; Hildyard *et al.*, 2018): in these studies, *SDHA* was very stable in canine muscle, but showed prominent muscle and disease-specific expression in murine muscle. This supports that specific reference genes for specific tissues should be identified for each individual model organism, rather than extrapolating between them.

The samples used to prepare our dataset were selected to offer relatively comprehensive coverage of potential variability and included healthy and dystrophic samples taken from different individuals, and from multiple brain regions. However, a number of limitations must be acknowledged: samples were derived from only five healthy and five dystrophic individuals for each studied species (dog and mouse), with six brain regions studied in the canine brain, and three in the murine brain. We have not assessed alternative DMD models, such as the *mdx ^3cv^* and *mdx ^4cv^* mouse line nor the Golden Retriever Muscular Dystrophy (GRMD) model (animal models with dystrophin mutations at different sites) and our samples do not assess changes with ageing. The overall transcriptional stability revealed by our work however, and the absence of marked differences in expression between healthy and dystrophic brains, suggests that our high scoring candidates are likely to be broadly applicable, but future studies utilising alternative brain regions, animal models or ages would nevertheless be of interest.

## Conclusion

In this study we have identified reference genes suitable for use in brain tissue from two important animal models of DMD. In the DE50-MD dog, *SDHA, UBC* and *YWHAZ* were identified as suitable for normalisation, while in the *mdx* mouse, the top scoring genes were *GAPDH, RPL13A* and *CYC1*. These reference genes will facilitate future work aiming to identify disease-associated genes and so improve understanding of the spectrum of neurodevelopmental and cognitive deficits identified in patients with DMD. A similar panel of reference genes appropriate for use in human samples (regardless of patient age, individual genetic background and disease status) would be of considerable benefit to the field by enabling comparison of independent studies and trials.

## Data availablity

### Underlying data

Figshare: Dog Brain qPCR data. https://doi.org/10.6084/m9.figshare.14135804.v2 ( Crawford *et al.*, 2021a).

This project contains the following underlying data:

- Raw qPCR data from canine brains (samples were assessed in triplicate; individual readings and mean sample Cq are provided).

Figshare: Mouse Brain qPCR data. https://doi.org/10.6084/m9.figshare.14135660.v2 ( Crawford *et al.*, 2021b).

This project contains the following underlying data:

- Raw qPCR data from murine brains (samples were assessed in triplicate; individual readings and mean sample Cq are provided).

### Extended data

Figshare: Supplementary information: qPCR Reference Genes Manuscript. https://doi.org/10.6084/m9.figshare.14135660.v2 ( Crawford *et al.*, 2021c).

This project contains the following extended data:

- Supplementary Table 1: Cq values for 9 candidate reference genes for canine brain samples.
- Supplementary Table 2: Cq values for 11 candidate reference genes for murine brain samples.
- Supplementary Table 3: geNorm algorithm results for the full canine dataset or specified subgroups showing reduction in pairwise variation with additional reference genes
- Supplementary Table 4: geNorm algorithm results for the full murine dataset or specified subgroups showing reduction in pairwise variation with additional reference genes
- Supplement figure 1: A plot of the global Bestkeeper against *UBC* or *HRPT1*.
- Supplementary figure 2: *18S, ZFP91*, *MON2* and *UBC* expression normalised to the geometric mean of *GAPDH, RPL13A* and *CYC1*.

Data are available under the terms of the Creative Commons Attribution 4.0 International license (CC-BY 4.0).
