# Supplementary material for: Identification of quantitative polymerase chain reaction reference genes suitable for normalising gene expression in the brain of normal and dystrophic mice and dogs
Source: Wellcome Open Res. 2023 May 5;6:84. Originally published 2021 Apr 16. [Version 2] doi: 10.12688/wellcomeopenres.16707.2 (PMC10628364; doi:10.12688/wellcomeopenres.16707.2)
Supplement: Peer review history

# Reviewer report: Reviewer response for version 2

**DOI:** 10.21956/wellcomeopenres.21527.r68869

**Published:** 2023-11-06

**Authors:** Hernan Gonorazky

**Affiliations:** 1 The Hospital for Sick Children, University of Toronto, Toronto, Canada

This manuscript delves into the crucial aspect of selecting appropriate housekeeping genes for RT-qPCR experiments, aiming to enable the normalization of gene expression data for dystrophin gene. Dystrophinopathy results in behavioural and cognitive impairments, which restrict patients from active participation in society. It is important to understand the different isoforms of the dystrophin gene and its expression in the brain. The housekeeping genes identified in this manuscript play a pivotal role in advancing research concerning dystrophic brains.

 I agree with the authors that different ages of the models required to be studied and it has been described in the paper .

 I do not have anything else to add.

Is the work clearly and accurately presented and does it cite the current literature?

Yes

If applicable, is the statistical analysis and its interpretation appropriate?

I cannot comment. A qualified statistician is required.

Are all the source data underlying the results available to ensure full reproducibility?

Yes

Is the study design appropriate and is the work technically sound?

Yes

Are the conclusions drawn adequately supported by the results?

Yes

Are sufficient details of methods and analysis provided to allow replication by others?

Yes

Reviewer Expertise:

Neuromuscular disorders Clinical in humnas

I confirm that I have read this submission and believe that I have an appropriate level of expertise to confirm that it is of an acceptable scientific standard.

# Reviewer report: Reviewer response for version 2

**DOI:** 10.21956/wellcomeopenres.21527.r56981

**Published:** 2023-05-23

**Authors:** Hui Wang

**Affiliations:** 1 Department of Pathology and Laboratory Medicine, Perelman School of Medicine, University of Pennsylvania, Philadelphia, Pennsylvania, USA

I have no further comments to make.

Is the work clearly and accurately presented and does it cite the current literature?

Yes

If applicable, is the statistical analysis and its interpretation appropriate?

Yes

Are all the source data underlying the results available to ensure full reproducibility?

Yes

Is the study design appropriate and is the work technically sound?

Yes

Are the conclusions drawn adequately supported by the results?

Partly

Are sufficient details of methods and analysis provided to allow replication by others?

Yes

Reviewer Expertise:

Genetics, bioinformatics

I confirm that I have read this submission and believe that I have an appropriate level of expertise to confirm that it is of an acceptable scientific standard.

# Reviewer report: Reviewer response for version 1

**DOI:** 10.21956/wellcomeopenres.18422.r55651

**Published:** 2023-03-27

**Authors:** Hui Wang

**Affiliations:** 1 Department of Pathology and Laboratory Medicine, Perelman School of Medicine, University of Pennsylvania, Philadelphia, Pennsylvania, USA

Reverse transcription quantitative real-time PCR (RT-qPCR) is used to study gene expression, but accurate assessment requires normalization of expression data using validated reference genes. The study aimed to identify a panel of suitable reference genes for normalizing gene expression in the brain of healthy and dystrophic dogs and mice. The study found that SDHA, UBC, and YWHAZ are suitable reference genes for normalizing gene expression in the healthy and dystrophic canine brain, while GAPDH, RPL13A, and CYC1 are suitable for the healthy and dystrophic murine brain. The findings could facilitate future research to study gene expression in the brains of normal and dystrophic dogs and mice.

 Overall, the methods, results and conclusions are well organized and clearly stated. However, I do have a few suggestions and questions:

1. The authors showed that SDHA, UBC, and YWHAZ are suitable reference genes for normalizing gene expression in the healthy and dystrophic canine brain, while GAPDH, RPL13A, and CYC1 are suitable for the healthy and dystrophic murine brain. It would be interesting to see the practical usage of those genes. For example, the authors could select a few known differentially expressed genes in dystrophic brains and see how different reference genes may give different results in terms of their expression in cases and controls.
2. The authors could explain more on why they choose to use the geometric mean of the top 3 gene to normalize three low scoring genes. Why not use more genes or select a best one?
3. In mice and dogs, the selected best references genes is quite different. Could authors discuss more about why that is the case? And how this difference between different organisms could help future study in humans?

Is the work clearly and accurately presented and does it cite the current literature?

Yes

If applicable, is the statistical analysis and its interpretation appropriate?

Yes

Are all the source data underlying the results available to ensure full reproducibility?

Yes

Is the study design appropriate and is the work technically sound?

Yes

Are the conclusions drawn adequately supported by the results?

Partly

Are sufficient details of methods and analysis provided to allow replication by others?

Yes

Reviewer Expertise:

Genetics, bioinformatics

I confirm that I have read this submission and believe that I have an appropriate level of expertise to confirm that it is of an acceptable scientific standard.

# Response

**Published:** 2023-04-27

**Authors:** Abbe Abbe

**Affiliations:** Royal Veterinary College, London, UK

*Thank you for your time reviewing our manuscript, please find our responses below.*

 Overall, the methods, results and conclusions are well organized and clearly stated. However, I do have a few suggestions and questions:

1. The authors showed that SDHA, UBC, and YWHAZ are suitable reference genes for normalizing gene expression in the healthy and dystrophic canine brain, while GAPDH, RPL13A, and CYC1 are suitable for the healthy and dystrophic murine brain. It would be interesting to see the practical usage of those genes. For example, the authors could select a few known differentially expressed genes in dystrophic brains and see how different reference genes may give different results in terms of their expression in cases and controls.

*Thank you for this suggestion. One of the more challenging aspects of the study of dystrophic brains is that comparatively little is currently known, particularly at the level of gene expression: dystrophin is known to be expressed in an isoform-specific manner within specific locations (and more broadly during development), but the longer-term effects of dystrophin deficiency remain contentious. These issues are further compounded by the nature of the brain itself, where much is governed at the level of architecture rather than gene expression: subtle differences in connectivity might elicit marked phenotypic alterations with only modest effects at the transcriptional level. Our data here, notably, supports a model whereby transcriptional differences between healthy and dystrophic brains are markedly less prominent than differences between individual brain regions (regardless of genotype).* *Needless to say, before one can study potential disease-associated transcriptional changes (even speculatively), it is necessary to establish a panel of suitable reference genes, and the methods used within this manuscript are established, well-validated approaches that allow this determination to be made a priori, without recourse to established differentially expressed genes for corroboration.* *As there are no canonically “differentially expressed genes” currently established in dystrophic brains (beyond dystrophin itself, and even here isoform expression patterns may vary by mutation), the essential underlying purpose of the work shown here is to facilitate the validation of any DEGs we might identify via other means (such as RNAseq).* *It might interest the reviewer to note that we have already used these genes to assess expression of dystrophin isoforms within healthy and DE50-MD canine brains, and here their use allowed detection of even subtle gene expression changes (implying our methods have indeed identified viable references).*

1. The authors could explain more on why they choose to use the geometric mean of the top 3 gene to normalize three low scoring genes. Why not use more genes or select a best one?

*As noted in the introduction, in accordance with the MIQE guidelines, any qPCR normalisation should endeavour to use multiple reference genes: a single gene is highly vulnerable to even modest variation (biological, experimental or technical). Two is considered the bare minimum for valid interpretation, while three renders normalisation highly robust to stochastic noise (an inherent risk in gene expression studies). In most cases, little additional stability is gained by increasing the panel from three to four (and indeed geNorm measures this empirically), and addition of yet more genes increasingly runs the risk of making the normalisation _less_ stable.* *Accordingly, we have avoiding implying any single gene is “best”, and instead have identified the top three high-scoring candidates, and used these three to generate a combined “normalisation factor (NF)”*

*The geometric mean is the correct averaging manipulation to combine multiple reference genes that have been linearised to relative quantities (RQ) as here. Linearised qPCR data follows a lognormal distribution and thus should be averaged in a manner appropriate for such distributions (conversely, for generating means using raw Cq values, as employed in ddCt methods, a simple arithmetic mean is appropriate).*

1. In mice and dogs, the selected best references genes is quite different. Could authors discuss more about why that is the case? And how this difference between different organisms could help future study in humans?

*There are multiple reasons why this might be the case: species differences are more common than not, and expecting similar genes to be appropriate across species boundaries is ill-advised. Our work highlights exactly such an example. Furthermore, size likely plays a major role, as does cognitive capacity, both of which lead to differences in respective relative volumes of given brain regions, and their specialisations. Moreover, we studied different regions in each species: while the large size of canine brains permits greater regional division, the small size of murine brains renders comparable detail prohibitive. Additionally, while murine brains can be collected rapidly after sacrifice, canine brains necessitate longer delays between death and sample collection (see methods*). *Finally, our panels of reference genes were not identical between species: both panels were taken from the geNorm/geNormPLUS reference gene sets (primerdesign), and were based on datamining of a large number of public microarray datasets: if a gene is present within the canine panel, that gene has exhibited high stability across multiple datasets. If a gene is not present, likely that gene has not demonstrated stability, and would therefore make for a poor candidate anyway. We endeavoured to ensure significant overlap between our lists, allowing us to potentially identify ‘universal’ reference genes, but our data shows this is not the case: the same gene that performs strongly in one species might perform poorly in another.* *With respect to humans, we would advise against drawing unwarranted conclusions: human brains are larger than canine brains, but humans are also genetically closer to mice than to dogs. We would strongly suggest that any investigators hoping to normalise gene expression in human brains would employ a methodology similar to ours, and identify suitable reference genes a priori.*

# Reviewer report: Reviewer response for version 1

**DOI:** 10.21956/wellcomeopenres.18422.r43516

**Published:** 2021-05-04

**Authors:** Maaike van Putten

**Affiliations:** 1 Department of Human Genetics, Leiden University Medical Center, Leiden, The Netherlands

This manuscript focuses on the important aspect of utilizing suitable housekeeping genes for RT-qPCR experiments, to allow for the normalization of gene expression data. The dystrophin gene has several promotors that not only give rise to the full-length isoform expressed in muscle, but also to several brain specific isoforms. Consequently, dystrophinopathy leads to behavioural and intellectual impairments, limiting patients to fully participate in society. Investigating brain pathology in murine and canine DMD models is therefore vital. The identified housekeeping genes that are described in this manuscript will facilitate research focused on dystrophic brains. The manuscript reads well, is novel and will appeal to the readers of this journal. I do however have several points that need to be addressed:

**Major point:**

 Authors have selected three brain regions to study in mice (cortex, cerebellum and brain stem), and six brain regions for the dog (olfactory bulbs, frontal cortex, temporal cortex, occipital cortex, brainstem, cerebellum). The reasoning for selecting these particular regions is lacking in the introduction section and should be added. Even though the regions that were selected are of importance, I do wonder why authors refrained from including the hippocampus and/or amygdala. Dystrophin expression is high in these particular regions (Doorenweerd *et al.* 2019, Sci Rep), and many of the behavioral deficits observed in patients/mice, are linked to the lack of dystrophin in these particular regions. I would strongly advise authors to add at least one of these highly dystrophin-abundant regions to this study.

**Minor points:**

**Introduction section**

 In the introduction section, references are lacking for the two genes that were selected based on pig data ( *ACTB, YWHAZ*).

 In the same sentence, they refer to HRPT found in murine, while HRPT1 is consistently used throughout the manuscript. Also, both SHDA and SDHA are used, which is likely a typo. Throughout the manuscript also RPL13A and RPL13a are used. In table 6, typo in ACTB8. Please double check the spelling of gene names, and strive for consistency both in text and tables/figs. Maybe consider underlying in tables instead of italics, such that gene names can be correctly written.

 I also wonder whether the reference to the dog muscle samples is required? The genes specified are already mentioned in the other selections based on the human brain (SDHA) and mouse brain (HPRT1 and RPL13a).

 While authors have identified YWHAZ also based on rodent data, they refrain from testing this in the murine samples. Please explain why this was left out.

 Based on which data were ZFP91 and MON2 selected for the murine samples?

 Authors write gene names with capitals; this only applies when referring to the human genome, but not to the i.e. the murine genome (here only the first letter is a capital). Please revise accordingly.

 Figure 1 + legend: There is a red line spelling check underneath Spectrin; please remove.

 Would be good to write Dp40 in the figure as well i.e Dp71/40. Last sentence exon 23 is written with capital E, better to replace it with an e, to match canine exon 50.

**Methods**

 I do wonder what authors refer to with the last bit in between brackets of the following sentence: “A total of 10 mice and 10 dogs were used in this study (experimental unit: individual animal)”

 The authors provide the sequence of primers used for HPRT1, but in front of the sequence, the name HPSF is written. This seems like another gene?

 It would be appreciated if authors could provide the sequences for all primers used in a table.

 Authors specifically indicate that for the Gapdh primers design spanned intronic regions (F primer in one exons and R primer in the neighboring exon). This is indeed the accurate method for primer design to prevent amplification of genomic DNA. I do however wonder whether this was true for all other primers? If so, please be more clear about this instead of specifying this for only one primer pair.

 It would be nice if the order of genes provided in table 1 and Figure 2 would match.

**Results**

 Figure 4: It would be nice if the scaling of the axis is similar between the three graphs, as they now deviate in the number of subdivisions, being only more detailed for the DMD dogs.

 While in table 2 and 6, >1 genes are just combined in the table box, for table 4 and 9 the word ‘and’ is used. Try to be consistent.

 In the legend of figure 7, the authors write: “Normalisation of B2M revealed elevated expression within the olfactory bulbs.” In the figure WT levels are somewhat higher than those of DMD dogs; is this significant? Differences for this region (being elevated in DMD dogs) were found for Actb, although most likely not reaching significance due to high variability. Please rephrase, or add significance indication in figure if indeed found to be sig. elevated.

**Discussion**

 “A recent study reported YWHAZ, UBC and SDHA (alongside HMBS) to be a suitable reference ….. and is written in italics.

 Authors describe many factors that could influence the high variability seen between individual dogs. I however miss the aspect of age. Although it has not been studied to date in dogs, it is known that brain pathology progresses with age in mice (Bagdatlioglu et al. NMD 2020). Given that the age range was between 6-19 months in dogs, I wonder whether authors looked into whether the covariate age does affect results and whether variation was higher in DMD dogs compared to WT.

**Supplementary files**

 I would encourage authors to double check labeling of their excel files and data, as there are inconsistencies: File entitled mouse brain GAPDH and S18….. has in sheet 1 labeling SDHA and UBC, while in sheet 2 this is GADPH and S18. This is just an example; please check all, since it happens with many, if not all. I would also be good to add WT and MDX + brain regions in sheet two next to the Cq values, so that one does not have to constantly switch between sheet 1 and 2 to understand the data and its labeling.

 The link provided under the caption data availability; extended data referring to the figures is the same as for the excel files of the murine qPCR data. Only the link provided in the references section (Crawford et al., 2021c) is correct. Please check all links.

 The way in which words are interrupted due to lack of space should be addressed in the tables.

 Sup table 3+4; tables read better if values that match the 0.2 criteria are written in bold.

Is the work clearly and accurately presented and does it cite the current literature?

Yes

If applicable, is the statistical analysis and its interpretation appropriate?

Yes

Are all the source data underlying the results available to ensure full reproducibility?

Yes

Is the study design appropriate and is the work technically sound?

Yes

Are the conclusions drawn adequately supported by the results?

Yes

Are sufficient details of methods and analysis provided to allow replication by others?

Partly

Reviewer Expertise:

Duchenne muscular dystrophy

I confirm that I have read this submission and believe that I have an appropriate level of expertise to confirm that it is of an acceptable scientific standard, however I have significant reservations, as outlined above.

# Response

**Published:** 2023-04-27

**Authors:** Abbe Abbe

**Affiliations:** Royal Veterinary College, London, UK

**Major point:**

 Authors have selected three brain regions to study in mice (cortex, cerebellum and brain stem), and six brain regions for the dog (olfactory bulbs, frontal cortex, temporal cortex, occipital cortex, brainstem, cerebellum). The reasoning for selecting these particular regions is lacking in the introduction section and should be added. Even though the regions that were selected are of importance, I do wonder why authors refrained from including the hippocampus and/or amygdala. Dystrophin expression is high in these particular regions (Doorenweerd *et al.* 2019, Sci Rep), and many of the behavioral deficits observed in patients/mice, are linked to the lack of dystrophin in these particular regions. I would strongly advise authors to add at least one of these highly dystrophin-abundant regions to this study.

*All canine samples used in this study were taken from previously collected tissue archives (collected as part of a larger disease-characterisation study). While greater subdivision of brain regions would be advantageous, the brain regions described in the manuscript were available at the time of initiating this study, and hence were utilised in preference to the breeding and use of animals specifically for this study. This information has now been included in the introduction. Given our work suggests that a common panel of reference genes can be used to compare brain regions as different as cortex, brainstem and cerebellum, the latter of which exhibits high expression of multiple dystrophin isoforms, we do not anticipate inclusion of additional brain regions would substantially alter our findings (and concomitantly, the genes suggested are likely to be valid references for other canine brain regions).* *We fully acknowledge that the hippocampus and amygdala would be highly interesting and valuable regions to study and we thank the reviewer for suggesting this: we will endeavour to collect these additional brain regions in future separately funded studies.*

**Minor points:**

**Introduction section**

 In the introduction section, references are lacking for the two genes that were selected based on pig data ( *ACTB, YWHAZ*).

*We have been unable to identify the original reference for this and so have deleted this statement from the manuscript.*

 In the same sentence, they refer to HRPT found in murine, while HRPT1 is consistently used throughout the manuscript. Also, both SHDA and SDHA are used, which is likely a typo. Throughout the manuscript also RPL13A and RPL13a are used. In table 6, typo in ACTB8. Please double check the spelling of gene names, and strive for consistency both in text and tables/figs. *Thank you- these have now been corrected.*    

 Maybe consider underlying in tables instead of italics, such that gene names can be correctly written.

*This has been modified as suggested.*

 I also wonder whether the reference to the dog muscle samples is required? The genes specified are already mentioned in the other selections based on the human brain (SDHA) and mouse brain (HPRT1 and RPL13a).

*Our intention here is to highlight that some genes (HPRT1 and RPL13a in particular) often appear to be strong references in multiple tissue types, and even across species. While we would not suggest these genes are “universal” in any sense, we would nevertheless encourage other authors to include such genes in candidate panels when establishing appropriate references for new scenarios.*

 While authors have identified YWHAZ also based on rodent data, they refrain from testing this in the murine samples. Please explain why this was left out.

*This was an arbitrary decision based on 11 genes being deemed sufficient, as well as availability of previously used and validated primers.*

 Based on which data were ZFP91 and MON2 selected for the murine samples?

*This information has now been added into the manuscript.*

 Authors write gene names with capitals; this only applies when referring to the human genome, but not to the i.e. the murine genome (here only the first letter is a capital). Please revise accordingly.

*We assume the reviewer is referring to Sundberg and Schofield, and/or the specification requested by certain journals. We note that this nomenclature scheme is primarily intended to alleviate ambiguity specifically in scenarios comparing humans and rodents (for example, a mouse carrying both a human transgene and its own endogenous mouse ortholog). We are not making such comparisons, nor is any ambiguity consequently likely to result from our chosen scheme (which we have moreover employed routinely in other manuscripts, again under scenarios where human/mouse comparisons are not applicable).* *We further note that no similar established nomenclature guidelines appear to exist for canine genes, making the widespread adoption of this nomenclature scheme somewhat contentious: would the reviewer prefer canine genes to follow murine convention or human/NHP convention?* *For the sake of brevity, simplicity, and consistency with established published work, we would prefer to retain the nomenclature scheme as it presently appears.*  

 Figure 1 + legend: There is a red line spelling check underneath Spectrin; please remove.

*Thank you, this has been corrected.*

 Would be good to write Dp40 in the figure as well i.e Dp71/40. *Excellent suggestion, thank you.*

*This has been added as suggested.*  

 Last sentence exon 23 is written with capital E, better to replace it with an e, to match canine exon 50.

*This has been corrected.*

**Methods**

 I do wonder what authors refer to with the last bit in between brackets of the following sentence: “A total of 10 mice and 10 dogs were used in this study (experimental unit: individual animal)”

*In our experience, some confusion often surrounds the precise definition of an experimental unit, and we (in accordance with ARRIVE suggestions) included this statement to render any such ambiguity moot (i.e. regardless of number of brain regions from an individual, this remains N=1). We now appear to have inadvertently introduced confusion by specifying this and so are happy to remove this line.*

 The authors provide the sequence of primers used for HPRT1, but in front of the sequence, the name HPSF is written. This seems like another gene?

*This is the name for the primers used by the authors in the cited reference, which we have preserved for consistency with that work. We understand HPSF to mean “HPrt1 pSeudogene Free”, as pseudogene amplification is a known hazard of some reference genes. We have now additionally included HRPT1 to avoid any confusion.*  

 It would be appreciated if authors could provide the sequences for all primers used in a table.

*The bulk of the primers used in this study are proprietary (taken from the primerdesign genorm set), and consequently we do not have these sequences. In accordance with the revised MIQE guidelines, anchor nucleotides and context lengths are regarded as acceptable substitutes for sequence data: we have provided references where such information can be found.*

 Authors specifically indicate that for the Gapdh primers design spanned intronic regions (F primer in one exons and R primer in the neighboring exon). This is indeed the accurate method for primer design to prevent amplification of genomic DNA. I do however wonder whether this was true for all other primers? If so, please be more clear about this instead of specifying this for only one primer pair.

*This has been clarified as suggested. The GAPDH primers were (as noted in the manuscript) designed specifically for this work, while the other primers are either from previously published work (HPRT1) or from the primerdesign genorm/genormPLUS sets.* *In most cases commercial primers from the genorm set do indeed span introns, however some are non-intron spanning. We have historically validated our methodology, and these primers, to confirm that any trace genomic DNA amplification will not meaningfully contribute to our measured values (references addressing this are cited within the manuscript).*

 It would be nice if the order of genes provided in table 1 and Figure 2 would match *This has been modified as suggested*.

**Results**

 Figure 4: It would be nice if the scaling of the axis is similar between the three graphs, as they now deviate in the number of subdivisions, being only more detailed for the DMD dogs.

*This has been modified as suggested.*

 While in table 2 and 6, >1 genes are just combined in the table box, for table 4 and 9 the word ‘and’ is used. Try to be consistent.

 “ *and” has been removed from tables 4 and 9*.

 In the legend of figure 7, the authors write: “Normalisation of B2M revealed elevated expression within the olfactory bulbs.” In the figure WT levels are somewhat higher than those of DMD dogs; is this significant? Differences for this region (being elevated in DMD dogs) were found for Actb, although most likely not reaching significance due to high variability. Please rephrase, or add significance indication in figure if indeed found to be sig. elevated.

*The figure legend referred to the fact that B2M expression appeared to be specifically enriched in the olfactory bulb as compared to other brain regions (regardless of genotype) – we have now clarified this in the manuscript.*

**Discussion**

 “A recent study reported YWHAZ, UBC and SDHA (alongside HMBS) to be a suitable reference ….. and is written in italics. #

*This has been corrected.*

 Authors describe many factors that could influence the high variability seen between individual dogs. I however miss the aspect of age. Although it has not been studied to date in dogs, it is known that brain pathology progresses with age in mice (Bagdatlioglu et al. NMD 2020). Given that the age range was between 6-19 months in dogs, I wonder whether authors looked into whether the covariate age does affect results and whether variation was higher in DMD dogs compared to WT.

*Given our small sample size and our use of dogs with very similar ages (only one dog was 6 months old and the remaining were 12-19 months) we did not investigate if age affected the data.  In our recently published study evaluating MRI findings in the DE50-MD canine brain, we did not see progression of gray matter atrophy over the study period of 14-33 months, but it would certainly be interesting to further investigate if age alters the brain pathology using techniques such as cognitive testing and histopathological analysis.*

# Response

**Published:** 2023-04-27

**Authors:** Abbe Abbe

**Affiliations:** Royal Veterinary College, London, UK

*We would like to thank the reviewer for their detailed review of our manuscript and their useful comments, suggestions and questions*.

 Regarding the supplementary files: File entitled mouse brain GAPDH and S18….. has in sheet 1 labeling SDHA and UBC, while in sheet 2 this is GADPH and S18. This is just an example; please check all, since it happens with many, if not all. I would also be good to add WT and MDX + brain regions in sheet two next to the Cq values, so that one does not have to constantly switch between sheet 1 and 2 to understand the data and its labeling.

*These have been checked.*

 The link provided under the caption data availability; extended data referring to the figures is the same as for the excel files of the murine qPCR data. Only the link provided in the references section (Crawford et al., 2021c) is correct. Please check all links

*These have been checked and corrected.*

   The way in which words are interrupted due to lack of space should be addressed in the tables.

*When the file is downloaded the words are displayed without interruption (this appearance is only when viewing the file on Figshare)*

 Sup table 3+4; tables read better if values that match the 0.2 criteria are written in bold.

*This would apply to all values and hence we have elected not to change this.*
